# Supplementary figures and images for: Diversity of rhizosphere and endophytic fungi in Atractylodes macrocephala during continuous cropping
Source: PeerJ. 2020 Apr 6;8:e8905. doi: 10.7717/peerj.8905 (PMC7144587; doi:10.7717/peerj.8905)

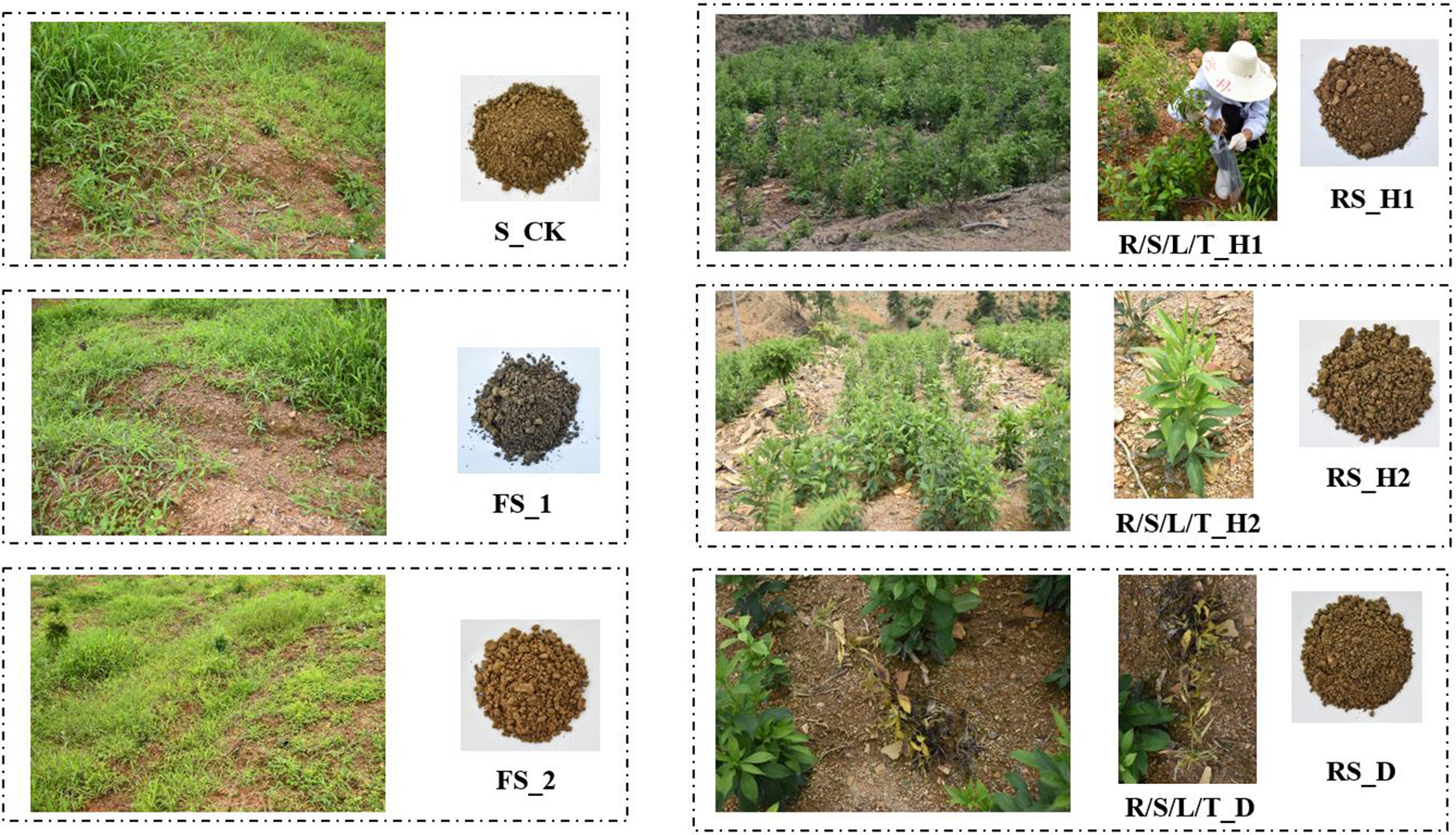

Supplement: Figure S1 — 1-year fallow soil (FS 1), 2-year fallow soil (FS 2), 1-year healthy rhizospheric soil (RS_H1), 2-year healthy rhizospheric soil (RS_H2), 2-year root-rot diseased rhizospheric soil (RS_D), blank control soil with no A. macrocephala planted (S_CK), 1-year-old healthy root/stem/leaf/tuber (R/S/L/T_H1), 2-year-old healthy root/stem/leaf/tuber (R/S/L/T_H2), 2-year-old root-rot diseased root/stem/leaf/tuber (R/S/L/T_D) [file peerj-08-8905-s001.png]
