# Supplementary material for: Diversity of rhizosphere and endophytic fungi in Atractylodes macrocephala during continuous cropping
Source: PeerJ. 2020 Apr 6;8:e8905. doi: 10.7717/peerj.8905 (PMC7144587; doi:10.7717/peerj.8905)
Supplement: Supplemental Information 2 — Organic matter (OM), total N (TN), hydrolysis N (HN), available P (AP), electrical conductivity (EC) [file peerj-08-8905-s002.docx]

Table S1 Spearman’s correlation coefficient between fungal community relative abundance at genus level and soil physicochemical factors

| Genus name | pH | OM | TN | HN | AP | EC | Hg | Cr |
| --- | --- | --- | --- | --- | --- | --- | --- | --- |
| *Fusarium* | -0.2970 | -0.3150 | -0.3980 | -0.5070 | -0.1120 | 0.2430 | 0.1860 | -0.4590 |
| Unclassified_f__norank_o__Pleosporales | 0.1790 | 0.0980 | -0.0090 | 0.2880 | 0.0650 | -0.0840 | -0.2620 | 0.3230 |
| Unclassified_k__Fungi | -0.0300 | 0.5190 | 0.4880 | 0.0670 | -0.5360 | -0.6040 | -0.4460 | -0.3040 |
| *Trichoderma* | 0.0260 | 0.0720 | 0.0480 | -0.2940 | -0.2790 | -0.4250 | -0.2990 | -0.3980 |
| *Talaromyces* | -0.1200 | 0.1550 | 0.0450 | -0.3640 | -0.4220 | -0.4060 | -0.3030 | -0.5140 |
| Unclassified_p__Ascomycota | 0.2180 | 0.0260 | 0.1190 | 0.2630 | 0.1290 | -0.1060 | -0.2240 | 0.3130 |
| Unclassified_f__Davidiellaceae | 0.1560 | -0.1370 | -0.1080 | 0.2360 | 0.5150 | 0.1700 | -0.0840 | 0.5850 |
| Unclassified_f__norank_o__Helotiales | -0.4810 | -0.1200 | -0.1890 | -0.2070 | -0.2750 | 0.2890 | 0.2910 | -0.3510 |
| *Alternaria* | -0.0200 | -0.1560 | -0.0970 | 0.1680 | 0.3880 | 0.2730 | 0.0540 | 0.4360 |
| Unclassified_f__Mycosphaerellaceae | 0.0950 | 0.0680 | 0.1370 | 0.3950 | 0.3330 | 0.1640 | -0.0260 | 0.5250 |
| *Uwebraunia* | 0.2350 | -0.0990 | 0.0700 | 0.4420 | 0.3650 | 0.2230 | 0.1000 | 0.5410 |
| *Penicillium* | -0.4070 | 0.1590 | 0.0470 | -0.5080 | -0.5510 | -0.2870 | -0.0550 | -0.7450 |
| *Cryptococcus* | -0.0920 | 0.1180 | 0.1870 | 0.1320 | 0.1290 | 0.0140 | -0.0320 | 0.1910 |
| Unclassified_f__Ceratobasidiaceae | 0.4950 | 0.1020 | -0.0180 | 0.3800 | 0.1030 | -0.2840 | -0.4290 | 0.4170 |
| *Phoma* | 0.3090 | -0.0570 | -0.0430 | 0.3270 | 0.4650 | -0.0140 | -0.2980 | 0.6240 |
| *Lophiostoma* | 0.3910 | 0.3660 | 0.2140 | 0.2070 | -0.1050 | -0.5320 | -0.6810 | 0.1620 |
| Unclassified_o__Pleosporales | 0.0810 | 0.2360 | 0.1350 | 0.3480 | 0.0310 | 0.0640 | -0.0860 | 0.3200 |
| *Mortierella* | -0.0340 | 0.3400 | 0.2530 | -0.2520 | -0.4710 | -0.6430 | -0.5280 | -0.4540 |
| Unclassified_c__Leotiomycetes | -0.1530 | -0.0070 | -0.1290 | -0.4380 | -0.1970 | -0.3110 | -0.2610 | -0.3670 |
| *Aspergillus* | 0.1040 | 0.2460 | 0.0780 | -0.2120 | -0.2840 | -0.5110 | -0.5090 | -0.3000 |
| *Cotylidia* | 0.2940 | -0.0660 | -0.1600 | -0.0970 | 0.1130 | -0.2310 | -0.3700 | 0.0680 |
| *Cladophialophora* | -0.3690 | 0.2700 | 0.1420 | -0.1210 | -0.6010 | -0.0550 | 0.0540 | -0.5080 |
| *Clonostachys* | -0.1040 | 0.0040 | -0.0870 | -0.3440 | -0.2640 | -0.1460 | -0.1530 | -0.4620 |
| *Phialophora* | -0.3650 | 0.2960 | 0.3360 | -0.2080 | -0.5680 | -0.3510 | -0.2000 | -0.5360 |
| Unclassified_f__Trichocomaceae | -0.4000 | 0.3360 | 0.3020 | -0.2490 | -0.6740 | -0.3190 | -0.0610 | -0.6570 |
| *Geminibasidium* | -0.2990 | 0.3390 | 0.2880 | -0.3250 | -0.6000 | -0.4540 | -0.1770 | -0.6660 |
| *Chalara* | 0.0040 | 0.2220 | 0.1190 | -0.1350 | -0.5010 | -0.2520 | -0.1050 | -0.4940 |
| Unclassified_o__Helotiales | -0.3900 | 0.1370 | 0.1080 | -0.3180 | -0.4970 | -0.1360 | -0.0380 | -0.6080 |
| *Chloridium* | 0.0580 | 0.2920 | 0.1820 | -0.2150 | -0.3870 | -0.6210 | -0.5530 | -0.3630 |
| *Acremonium* | 0.4870 | 0.3450 | 0.2890 | 0.0630 | 0.0560 | -0.8000 | -0.8660 | 0.1710 |
| *Sporobolomyces* | 0.1180 | -0.0250 | 0.0160 | 0.4850 | 0.2610 | 0.4400 | 0.2300 | 0.4890 |
| *Ilyonectria* | 0.2060 | -0.0600 | -0.1350 | -0.1650 | -0.0660 | -0.2830 | -0.4000 | -0.1360 |
| *Lectera* | 0.3050 | 0.2640 | 0.2960 | 0.5590 | -0.1800 | -0.0990 | -0.3140 | 0.2340 |
| Unclassified_c__Dothideomycetes | 0.1290 | 0.0220 | 0.1290 | 0.1960 | 0.2330 | -0.0400 | -0.1180 | 0.3280 |
| *Oidiodendron* | -0.2960 | 0.3900 | 0.3890 | -0.2130 | -0.6370 | -0.4380 | -0.1780 | -0.6360 |
| Unclassified_o__Leucosporidiales | -0.0900 | 0.3860 | 0.3290 | -0.2530 | -0.4920 | -0.5120 | -0.2720 | -0.5480 |
| *Phialocephala* | -0.1030 | 0.3100 | 0.2810 | -0.0170 | -0.5570 | -0.2130 | -0.1540 | -0.4710 |
| *Rhodosporidium* | 0.5800 | -0.0020 | 0.1330 | 0.5310 | 0.5310 | -0.1330 | -0.2740 | 0.7520 |
| Unclassified_o__Sporidiobolales | -0.0670 | -0.3580 | -0.2240 | 0.1030 | 0.4220 | 0.4820 | 0.3290 | 0.3450 |
| Unclassified_f__Glomeraceae | 0.3150 | -0.3800 | -0.3800 | -0.1370 | 0.4360 | -0.2650 | -0.3580 | 0.3300 |
| *Myrothecium* | 0.3030 | 0.5500 | 0.5770 | 0.3760 | -0.3200 | -0.6010 | -0.6100 | 0.0310 |
| *Cladosporium* | -0.1820 | -0.0090 | -0.0680 | -0.3760 | -0.1790 | -0.2800 | -0.1920 | -0.2980 |
| *Penicillifer* | -0.5580 | 0.0500 | -0.0220 | -0.5880 | -0.5220 | 0.0330 | 0.2650 | -0.8200 |
| *Microbotryozyma* | 0.1280 | -0.1440 | -0.0110 | 0.2460 | 0.3790 | 0.2810 | 0.2200 | 0.4430 |
| *Monographella* | 0.4250 | 0.4850 | 0.3880 | 0.3490 | -0.2440 | -0.5830 | -0.4980 | 0.0970 |
| Unclassified_f__Sordariaceae | -0.0620 | 0.2760 | 0.2200 | 0.0360 | -0.4210 | -0.2990 | -0.4160 | -0.2380 |
| Unclassified_f__Herpotrichiellaceae | 0.0970 | -0.0530 | -0.0990 | -0.1200 | -0.1450 | -0.2380 | -0.2740 | -0.1440 |
| *Thermomyces* | -0.0240 | 0.3640 | 0.3640 | -0.1600 | -0.2460 | -0.5640 | -0.5960 | -0.2400 |
| *Ophiostoma* | 0.0280 | 0.0360 | 0.0720 | 0.3280 | 0.0360 | 0.2760 | 0.3090 | 0.2130 |
| *Rhodotorula* | -0.3400 | -0.0490 | -0.0190 | 0.1800 | -0.0410 | 0.6050 | 0.5600 | 0.0270 |

-Organic matter (OM), total N (TN), hydrolysis N (HN), available P (AP), electrical conductivity (EC)
