# Supplementary material for: Diversity of rhizosphere and endophytic fungi in Atractylodes macrocephala during continuous cropping
Source: PeerJ. 2020 Apr 6;8:e8905. doi: 10.7717/peerj.8905 (PMC7144587; doi:10.7717/peerj.8905)
Supplement: Supplemental Information 3 — Organic matter (OM), total N (TN), hydrolysis N (HN), available P (AP), electrical conductivity (EC) [file peerj-08-8905-s003.docx]

Table S2 *P* values of Spearman’s correlation coefficient between fungal community relative abundance at genus level and soil physicochemical factors

| Genus name | pH | OM | TN | HN | AP | EC | Hg | Cr |
| --- | --- | --- | --- | --- | --- | --- | --- | --- |
| *Fusarium* | 0.2310 | 0.2030 | 0.1020 | 0.0320 | 0.6600 | 0.3320 | 0.4600 | 0.0550 |
| Unclassified_f__norank_o__Pleosporales | 0.4780 | 0.6990 | 0.9710 | 0.2470 | 0.7980 | 0.7420 | 0.2930 | 0.1910 |
| Unclassified_k__Fungi | 0.9060 | 0.0270 | 0.0400 | 0.7910 | 0.0220 | 0.0080 | 0.0640 | 0.2190 |
| *Trichoderma* | 0.9180 | 0.7770 | 0.8500 | 0.2360 | 0.2630 | 0.0790 | 0.2290 | 0.1020 |
| *Talaromyces* | 0.6360 | 0.5380 | 0.8600 | 0.1380 | 0.0810 | 0.0950 | 0.2220 | 0.0290 |
| Unclassified_p__Ascomycota | 0.3850 | 0.9190 | 0.6390 | 0.2910 | 0.6100 | 0.6750 | 0.3710 | 0.2060 |
| Unclassified_f__Davidiellaceae | 0.5370 | 0.5870 | 0.6690 | 0.3450 | 0.0290 | 0.4990 | 0.7410 | 0.0110 |
| Unclassified_f__norank_o__Helotiales | 0.0430 | 0.6350 | 0.4520 | 0.4100 | 0.2690 | 0.2450 | 0.2420 | 0.1530 |
| *Alternaria* | 0.9380 | 0.5370 | 0.7020 | 0.5040 | 0.1110 | 0.2740 | 0.8320 | 0.0710 |
| Unclassified_f__Mycosphaerellaceae | 0.7080 | 0.7890 | 0.5890 | 0.1050 | 0.1770 | 0.5160 | 0.9180 | 0.0250 |
| *Uwebraunia* | 0.3490 | 0.6970 | 0.7820 | 0.0660 | 0.1360 | 0.3730 | 0.6940 | 0.0200 |
| *Penicillium* | 0.0940 | 0.5280 | 0.8540 | 0.0310 | 0.0180 | 0.2490 | 0.8290 | 0.0000 |
| *Cryptococcus* | 0.7170 | 0.6420 | 0.4580 | 0.6010 | 0.6100 | 0.9550 | 0.9000 | 0.4480 |
| Unclassified_f__Ceratobasidiaceae | 0.0370 | 0.6870 | 0.9420 | 0.1200 | 0.6830 | 0.2530 | 0.0760 | 0.0850 |
| *Phoma* | 0.2130 | 0.8230 | 0.8640 | 0.1850 | 0.0520 | 0.9550 | 0.2310 | 0.0060 |
| *Lophiostoma* | 0.1080 | 0.1360 | 0.3940 | 0.4110 | 0.6770 | 0.0230 | 0.0020 | 0.5200 |
| Unclassified_o__Pleosporales | 0.7510 | 0.3470 | 0.5920 | 0.1570 | 0.9030 | 0.8010 | 0.7350 | 0.1950 |
| *Mortierella* | 0.8950 | 0.1680 | 0.3100 | 0.3130 | 0.0490 | 0.0040 | 0.0240 | 0.0580 |
| Unclassified_c__Leotiomycetes | 0.5440 | 0.9770 | 0.6090 | 0.0690 | 0.4340 | 0.2080 | 0.2960 | 0.1340 |
| *Aspergillus* | 0.6830 | 0.3240 | 0.7590 | 0.3980 | 0.2540 | 0.0300 | 0.0310 | 0.2260 |
| *Cotylidia* | 0.2360 | 0.7930 | 0.5260 | 0.7010 | 0.6550 | 0.3560 | 0.1310 | 0.7900 |
| *Cladophialophora* | 0.1320 | 0.2790 | 0.5750 | 0.6320 | 0.0080 | 0.8290 | 0.8320 | 0.0310 |
| *Clonostachys* | 0.6800 | 0.9870 | 0.7330 | 0.1620 | 0.2900 | 0.5630 | 0.5430 | 0.0530 |
| *Phialophora* | 0.1360 | 0.2340 | 0.1720 | 0.4060 | 0.0140 | 0.1530 | 0.4270 | 0.0220 |
| Unclassified_f__Trichocomaceae | 0.1000 | 0.1730 | 0.2230 | 0.3190 | 0.0020 | 0.1970 | 0.8110 | 0.0030 |
| *Geminibasidium* | 0.2280 | 0.1680 | 0.2470 | 0.1880 | 0.0090 | 0.0580 | 0.4810 | 0.0030 |
| *Chalara* | 0.9890 | 0.3760 | 0.6390 | 0.5930 | 0.0340 | 0.3120 | 0.6800 | 0.0370 |
| Unclassified_o__Helotiales | 0.1090 | 0.5890 | 0.6710 | 0.1990 | 0.0360 | 0.5920 | 0.8800 | 0.0070 |
| *Chloridium* | 0.8180 | 0.2390 | 0.4700 | 0.3920 | 0.1130 | 0.0060 | 0.0170 | 0.1390 |
| *Acremonium* | 0.0400 | 0.1610 | 0.2440 | 0.8040 | 0.8240 | 0.0000 | 0.0000 | 0.4970 |
| *Sporobolomyces* | 0.6400 | 0.9230 | 0.9500 | 0.0410 | 0.2950 | 0.0680 | 0.3580 | 0.0390 |
| *Ilyonectria* | 0.4130 | 0.8120 | 0.5930 | 0.5130 | 0.7930 | 0.2550 | 0.1000 | 0.5910 |
| *Lectera* | 0.2190 | 0.2900 | 0.2330 | 0.0160 | 0.4740 | 0.6950 | 0.2040 | 0.3490 |
| Unclassified_c__Dothideomycetes | 0.6090 | 0.9320 | 0.6090 | 0.4360 | 0.3520 | 0.8740 | 0.6420 | 0.1830 |
| *Oidiodendron* | 0.2340 | 0.1100 | 0.1110 | 0.3960 | 0.0040 | 0.0690 | 0.4800 | 0.0050 |
| Unclassified_o__Leucosporidiales | 0.7240 | 0.1130 | 0.1830 | 0.3120 | 0.0380 | 0.0300 | 0.2750 | 0.0180 |
| *Phialocephala* | 0.6860 | 0.2110 | 0.2580 | 0.9480 | 0.0160 | 0.3970 | 0.5410 | 0.0490 |
| *Rhodosporidium* | 0.0120 | 0.9920 | 0.5990 | 0.0230 | 0.0230 | 0.5990 | 0.2720 | 0.0000 |
| Unclassified_o__Sporidiobolales | 0.7910 | 0.1440 | 0.3720 | 0.6860 | 0.0810 | 0.0430 | 0.1830 | 0.1610 |
| Unclassified_f__Glomeraceae | 0.2040 | 0.1200 | 0.1200 | 0.5870 | 0.0700 | 0.2880 | 0.1450 | 0.1810 |
| *Myrothecium* | 0.2220 | 0.0180 | 0.0120 | 0.1240 | 0.1950 | 0.0080 | 0.0070 | 0.9030 |
| *Cladosporium* | 0.4700 | 0.9710 | 0.7880 | 0.1250 | 0.4770 | 0.2600 | 0.4460 | 0.2300 |
| *Penicillifer* | 0.0160 | 0.8440 | 0.9300 | 0.0100 | 0.0260 | 0.8970 | 0.2880 | 0.0000 |
| *Microbotryozyma* | 0.6130 | 0.5690 | 0.9650 | 0.3250 | 0.1210 | 0.2580 | 0.3800 | 0.0660 |
| *Monographella* | 0.0780 | 0.0410 | 0.1110 | 0.1560 | 0.3300 | 0.0110 | 0.0350 | 0.7020 |
| Unclassified_f__Sordariaceae | 0.8060 | 0.2680 | 0.3810 | 0.8860 | 0.0820 | 0.2280 | 0.0860 | 0.3410 |
| Unclassified_f__Herpotrichiellaceae | 0.7010 | 0.8350 | 0.6950 | 0.6350 | 0.5660 | 0.3410 | 0.2700 | 0.5690 |
| *Thermomyces* | 0.9250 | 0.1380 | 0.1380 | 0.5250 | 0.3250 | 0.0150 | 0.0090 | 0.3380 |
| *Ophiostoma* | 0.9130 | 0.8870 | 0.7750 | 0.1840 | 0.8870 | 0.2680 | 0.2130 | 0.3970 |
| *Rhodotorula* | 0.1680 | 0.8470 | 0.9410 | 0.4750 | 0.8730 | 0.0080 | 0.0160 | 0.9150 |

-Organic matter (OM), total N (TN), hydrolysis N (HN), available P (AP), electrical conductivity (EC)
